# Surfactant Protein A Binds Flagellin Enhancing Phagocytosis and IL-1β Production

**DOI:** 10.1371/journal.pone.0082680

**Published:** 2013-12-02

**Authors:** Anastasia K. Ketko, Chinhong Lin, Bethany B. Moore, Ann Marie LeVine

**Affiliations:** 1 Department of Pediatrics, Division of Neonatology, Division of Pediatric Critical Care Medicine, University of Michigan, Ann Arbor, Michigan, United States of America; 2 Department of Internal Medicine, University of Michigan, Ann Arbor, Michigan, United States of America; National Institute of Infectious Diseases, Japan

## Abstract

Surfactant protein A (SP-A), a pulmonary collectin, plays a role in lung innate immune host defense. In this study the role of SP-A in regulating the inflammatory response to the flagella of *Pseudomonas aeruginosa* (PA) was examined. Intra-tracheal infection of SP-A deficient (SP-A-/-) C57BL/6 mice with wild type flagellated PA (PAK) resulted in an increase in inflammatory cell recruitment and increase in pro-inflammatory cytokines IL-6 and TNF-α, which was not observed with a mutant pseudomonas lacking flagella (*fliC*). SP-A directly bound flagellin, via the N-linked carbohydrate moieties and collagen-like domain, in a concentration dependent manner and enhanced macrophage phagocytosis of flagellin and wild type PAK. IL-1β was reduced in the lungs of SP-A-/- mice following PAK infection. MH-s cells, a macrophage cell line, generated greater IL-1β when stimulated with flagellin and SP-A. Historically flagella stimulate IL-1β production through the toll-like receptor 5 (TLR-5) pathway and through a caspase-1 activating inflammasome pathway. IL-1β expression became non-detectable in SP-A and flagellin stimulated MH-s cells in which caspase-1 was silenced, suggesting SP-A induction of IL-1β appears to be occurring through the inflammasome pathway. SP-A plays an important role in the pathogenesis of PA infection in the lung by binding flagellin, enhancing its phagocytosis and modifying the macrophage inflammatory response.

## Introduction

Pulmonary surfactant protein (SP) A and SP-D belong to a group of collagen-like C-type or Ca^2+^-dependent lectins called collectins. The structure of SP-A consists of a glycosylated collagenous domain and a globular COOH-terminus (lectin) carbohydrate recognition domain (CRD). The CRD of SP-A recognizes oligosaccharide moieties on various pathogens to facilitate their clearance. SP-A enhances bacterial clearance by opsonization, enhancing phagocytosis, or by permeabilizing the bacterial membrane [1]. The role of SP-A in pathogen clearance is supported by the impaired bacterial clearance, exaggerated inflammatory response and increased oxidative injury observed in the lungs of SP-A deficient (SP-A-/-) mice following bacterial infection [2]. 

*Pseudomonas aeruginosa* (PA), a Gram negative bacteria, is an important pathogen in patients with cystic fibrosis lung disease or ventilator associated pneumonia. SP-A enhances PA clearance by either serving as an opsonin to promote PA phagocytosis [3] or by directly permeabilizing its membrane [1]. Following intra-tracheal PA infection, phagocytosis and bacterial clearance are impaired in SP-A-/- mice, which is associated with increased inflammation in the lungs [4]. Flagella play a role in PA pathogenicity, as it is a significant virulence factor [5] but also contributes to the pulmonary clearance of the organism [6]. SP-A enhances phagocytosis of wild type PA but not a PA flagellum-deficient mutant *flgE* [7].

Flagellin, the major structural protein of bacteria flagella, signals through Toll-like receptor (TLR)-5, stimulating the release of pro-inflammatory mediators such as tumor necrosis factor alpha (TNF-α), pro-interleukin (IL)-1β, IL-6 and IL-8. Flagellin functions as a ligand binding to TLR-5 resulting in nonopsonic phagocytosis of PA [6,8,9]. TLR-5 binding activates a MyD88-dependant signaling pathway resulting in activation of a variety of cellular signaling kinases including mitogen-activated protein (MAP) kinase p38, extracellular regulated kinase (ERK) and inhibitor of κB (IκB) kinase (IKK). IKK phosphorylates the inhibitory protein IκBα, resulting in its dissociation from NF-κB (nuclear factor kappa-light-chain-enhancer of activated B cells) which then translocates to the nucleus and binds the DNA, activating the transcription of genes for pro-inflammatory cytokines, such as pro-IL-1β. Pro-IL-1β can then be further processed into active IL-1β by a complex known as the inflammasome.

In addition to binding extracellular TLR-5, the flagellin protein stimulates inflammatory cytokine production through the intracellular NLRC4 (nucleotide-binding domain, leucine-rich repeat (NLR), caspase activation and recruitment domain (CARD)-containing 4) inflammasome pathway [10,11]. NLRC4, also known as IL-1β-converting enzyme (ICE) protease activating factor (IPAF), is important for PA flagellin-mediated activation of caspase-1 [12]. Flagellin is introduced into the cytosol, possibly via a type III secretion system (TTSS) or by the phagosome, and ultimately results in NLRC4 oligomerization, forming a large multi-protein complex known as an inflammasome, which is capable of activating IL-1β-converting enzyme known as caspase-1. Once activated, caspase-1 is capable of initiating a proinflammatory programmed cell death, known as pyroptosis, and also cleaves pro-IL-1β into its mature form.

Although there is clear evidence that phagocytosis of PA is impaired in the lungs of SP-A-/- mice, it remains unclear if this is due to mechanisms other than pathogen opsonization. The present study was undertaken to identify if SP-A binds to flagella as a mechanism to enhance PA phagocytosis and regulate the expression of pro-inflammatory cytokines in the lung in response to PA infection.

## Materials and Methods

### Ethics Statement

Animals were housed and studied under the approval and supervision of the University of Michigan’s University Committee on Use and Care of Animals (UCUCA, protocol number: PRO00004733), and in concordance with Unit for Laboratory Animal Medicine (ULAM) guidelines, in the animal facilities of the University of Michigan. University of Michigan’s UCUCA received AAALAC (Council on Accreditation of the Association for Assessment and Accreditation of Laboratory Animal Care) International approval on 01/27/12, USDA number 34-R-0001 (PHS Assurance: A3114-01, Exp 09/30/15). Human SP-A was purified from bronchoalveolar lavage fluid obtained from patients with alveolar proteinosis at the University of Cincinnati, where a formal written waiver was approved by the University of Cincinnati Institutional Review Board (Federalwide Assurance (FWA) number: 00003152) deeming consent unnecessary due to routine discarding of the fluid.

### Animal Husbandry

The murine SP-A gene locus was targeted by homologous recombination as previously described, the lungs of SP-A-/- mice are lacking detectable SP-A [13]. SP-A-/- and SP-A+/+ mice were maintained in strain C57BL/6. Eight sex-matched male and female mice of approximately 20-25 grams (42-56 days old) were used per experiment.

### Preparation of Bacteria and Flagellin

The wild type strain PAK, a commonly studied *P. aeruginosa* strain, and the PAK flagella-deficient *fliC* mutant were kindly provided by R. Ramphal (University of Florida, Gainesville, Fl). Bacteria for intra-tracheal inoculations were prepared as previously described [4]. Mice were infected with 1x10^8^ cfu of PAK or *fliC*. Bacteria for labeling were grown overnight in Luria-Bertani (LB) broth. The suspension was pelleted at maximum speed in a microfuge, resuspended in 0.9ml PBS (pH 7.2), and heated to 95°C for 10 minutes to kill the bacteria. The heat-killed bacteria were pelleted and resuspended in 1ml of 0.1M sodium carbonate (pH 9.0). Fluorescein isothiocyanate (FITC, Molecular Probes, Eugene, OR) was added as a 10mg/ml stock in dimethyl sulfoxide (DMSO) to a final concentration of 0.01mg/ml, and the suspension was incubated for 1 hour in the dark at room temperature with gentle agitation. Labeled bacteria were washed 4 times for 5 minutes with PBS (pH 7.2) to remove unconjugated fluorophore, diluted in PBS and then stored in aliquots of 100µl at -80°C.

PAK flagella were purified after overnight culture in LB broth. The broth was centrifuged at 4000rpm for 15 minutes and resuspended in 100ml of cold PBS containing 10mM MgCl_2_. The flagella were removed from the cells by shearing at low speed in a commercial blender for 2 minutes, followed by centrifugation at 10,000rpm for 30 minutes at 4°C. The flagella was then pelleted by ultracentrifugation at 10,000rpm for 5 hours at 4°C, resuspended in 1ml of PBS with 10mM MgCl_2_ and dialyzed in cold PBS containing 10mM MgCl2 at 4°C for 72 hours, using a Slide-a-Lyzer G2 dialysis cassette (Pierce, Rockford, IL). Protein purification was confirmed as a single band following the Coomassie blue technique of Bradford and protein concentration was determined using a bicinchoninic acid (BCA) protein assay (Pierce, Rockford, IL).

### SP-A Isolation

Human SP-A, obtained from patients with alveolar proteinosis, was purified by the 1-butanol extraction method of Haagsman et al. [14] and dissolved in NaHEPES (pH 7.2). Endotoxin contamination was not detected in SP-A preparations (< 0.06 EU/ml) using the Limulus Amoebocyte Lysate assay (Sigma, St. Louis, MO) according to the manufacturer's directions. Collagenase treated SP-A was prepared by incubation of 100µg of SP-A in 50mM Tris-HCL, 1mM EDTA, 100mM NaCl, 0.36mM CaCl_2_ (pH 7.4) buffer containing 187.5 units of collagenase (Sigma, St. Louis, MO) at 37°C for 72 hours. Deglycosylated SP-A (100µg) was prepared by incubating SP-A in 100mM NaH_2_PO_4_, 10mM Na_2_EDTA (pH 6.1) buffer containing 1000 units of peptide N-glycosidase F (New England Biolabs, Ipswich, MA) at 37°C for 72 hours. Samples were then centrifuged in a 30-kDa molecular mass cutoff Centricon column (Millipore, Billerica, MA) and digests were analyzed by SDS-PAGE on a 10-20% Tris-glycine gel. SP-A was fluorescently labeled by incubating 1µg of SP-A with 0.1M sodium bicarbonate (pH 9.0) and 0.03 µg of Alexa Fluor 488 carboxylic acid succinimidyl ester (Molecular Probes, Invitrogen, Grand Island, NY) at room temperature for 3 hours, followed by dialysis against 5mM HEPES buffer (pH 7.2) for 72 hours at 4°C to remove unbound label.

### Binding of SP-A to Flagellin

Flagellin (20µg/ml) was incubated overnight at 4°C in 100µl of 0.1M sodium bicarbonate (pH 8.5) in 96-well plates. The plates were washed twice with Tris-buffered saline (TBS, pH 7.4). The plates were then treated with 5% bovine serum albumin and 5% human albumin in TBS for 20 minutes at room temperature, followed by two additional washes with TBS. Alexa fluor 488-labeled SP-A, in concentrations from 2.5-20µg/ml, was added in 100µl of plating buffer (5mM HEPES, 1% bovine serum albumin in Tyrode's salt solution). The plates were incubated for 1 hour at 37°C, washed twice with TBS and the fluorescence determined by fluorometry. Additional experiments were performed with 20µg/ml of Alexa-fluor 488-labeled collagenase or N-glycosidase treated SP-A.

### Binding of SP-A to Scavenger Receptors

Protein purified from transfected High Five insect cells, using a ANTI-FLAG M2 Affinity gel (Sigma, St. Louis, MO), for the scavenger receptors macrophage scavenger receptors (SRA I and SRAII) or recombinant macrophage receptor with collagenous structure (MARCO) (R&D Systems, Minneapolis, MN) or bovine serum albumin as a control were bound to a 96 well plate overnight at 4°C in 50 mM Na_2_CO_3_, pH 9.5 at a concentration of 25 ng/ml. FITC labeled SP-A (prepared in a manner analogous to that described for bacteria above) at a concentration of 20µg/ml was then added to the wells and incubated for 2 h. Wells were washed 3 times with TBS buffer and fluorescence was determined by fluorometry as a measure of binding activity.

### Phagocytosis Assay

Phagocytosis by alveolar macrophages *in vivo* was measured by intra-tracheally infecting mice by catheter intubation, retracting the tongue to obstruct the esophagus, with FITC labeled PAK or *fliC*, followed by evaluation of internal cell-associated fluorescence by flow cytometry. Two hours after infection, macrophages from bronchoalveolar lavage (BAL) were resuspended in buffer (PBS, 0.2% BSA fraction V, 0.02% sodium azide) and then trypan blue (0.2mg/ml) was added to quench fluorescence of extracellular FITC for at least 15 minutes. Phagocytosis of flagellin was determined by isolating alveolar macrophages from SP-A+/+ and SP-A-/- mice, incubating the cells in buffer for 2 hours with FITC labeled flagellin, washing the cells and then trypan blue (0.2mg/ml) was added to quench fluorescence of extracellular FITC. Cell associated fluorescence for both experiments was measured on a FACScan flow cytometer, using CELLQuest software (Becton Dickinson, San Jose, CA). For each sample of macrophages, 20,000 cells were counted in duplicate and the results expressed as the percentage of macrophages with label, all macrophages were gated.

### MH-s Cell Stimulation

The MH-s murine alveolar macrophage cell line was purchased from the American Type Culture Collection (Manassas, VA). The cells were maintained in RPMI 1640 with 10% fetal bovine serum. The MH-s cells were adhered to six well tissue culture plates at a concentration of 1x10^6^ cells/ml overnight in 5% CO_2_ at 37°C. The following day the media was aspirated and 1ml of serum-free RPM1 1640 media, enriched with 2mM of CaCl, was added with either 2µg/ml of flagellin, 20µg/ml of SP-A or both the flagellin and SP-A. The cells incubated for 20 minutes or 60 minutes at 37°C. The media was then collected, centrifuged at 10,000rpm for 10 minutes and the supernatants were stored at -20°C. The cell pellets were washed with sterile PBS and cell lysis buffer was added followed by incubation of the pellet for 15 minutes on ice.

### Cytokine Production

Tumor necrosis factor alpha (TNF-α), interleukin (IL)-1β and IL-6 were quantitated in BAL fluid 6 and 24 hours following infection of the mice with PAK or *fliC*, or following an hour stimulation of MH-s cells. TNF-α, IL-1β, IL-6 were measured using murine sandwich ELISA kits (R&D systems, Minneapolis, MN) according to the manufacturer’s directions, which included measuring technical duplicates of each sample. All plates were read on a microplate reader (Molecular Devices, Menlo Park, CA) and analyzed with the use of a computer-assisted analysis program (Softmax; Molecular Devices). Only assays with standard curves with a calculated regression line value > 0.95 were accepted for analysis.

### Western Blot Analysis

MH-s cells were stimulated with flagellin (2µg/ml) or SP-A (20µg/ml) plus flagellin for 20 or 60 minutes in serum-free RPMI 1640 media. The cells were then lysed in lysis buffer (20mM Tris-HCL, pH 7.4, 150mM NaCl, 0.1% SDS, 1% NP-40, 1mM EDTA, 0.5mM DTT, 0.2mM PMSF). Protein was estimated using a detergent tolerant assay reagent system (Biorad, Hercules, CA) and equal amounts of protein were electrophoresed on an 8-16% Tris-glycine gel before being transferred onto a nitrocellulose membrane. The blot was sequentially probed, stripped and reprobed with antibodies to anti-phospho-p38 MAPK, p38MAPK and β-actin. Additional blots were probed with anti-phospho-ERK, anti-ERK, anti-phospho-IκB and anti IκB (all antibodies were from Cell Signaling Technology, Denver, MA). Primary blots were then incubated with the appropriate secondary antibody conjugated to horseradish peroxidase and developed by the ECL method. Western blot was also performed in separate experiments for caspase-1 (Santa Cruz, Santa Cruz, CA) on the MH-s cell lysates following stimulation. Membranes were rinsed and developed using enhanced chemiluminescence (ECL) detection reagents (Amersham, Arlington Heights, IL). Immunoreactive bands were identified by exposing the membranes to XAR film (Kodak, Rochester, NY). Densitometry was performed using the Image J free software program which analyzed the pixel area corresponding to each band.

### Caspase-1 siRNA

MH-s cells were transfected with On-Target plus Smart Pool siRNA Caspase-1 or On-Target plus Smart Pool control siRNA from Dharmacon (Lafayette, CO) via electroporation using the Mouse Macrophage Nucleofector Kit (Lonza, Basel, Switzerland). Briefly, MH-s cells were grown as described above and 1x10^6^ cells were resuspended in 100μL of Nucleofector solution. 100nM caspase-1 siRNA or control siRNA was added to the cell suspension and the cells underwent electroporation per manufacturer program using the Amaxa Nucleofector-II Device (Lonza, Basel, Switzerland). Transfection efficiency was determined by Western blot analysis of caspase-1 as described above. Cell viability at 48, 72 and 96 hours was determined microscopically after trypan blue staining. Following 48 hours of incubation cells were stimulated as described above and the media collected for the determination of cytokines.

### Statistical Methods

In circumstances where 2 groups were being compared to each other, student’s t-test was used to analyze the results; when 3 or more groups were being compared and data was normally distrubed, ANOVA was used with a post-hoc Bonferroni test. For some cytokine measurements, data were not assumed to be normally distributed; thus these data were analyzed by Kruskal-Walis ANOVA followed by a Dunn’s multiple comparison test. Findings were considered statistically significant at probability levels < 0.05. 

## Results

### Pulmonary Inflammation

**Figure 1:**
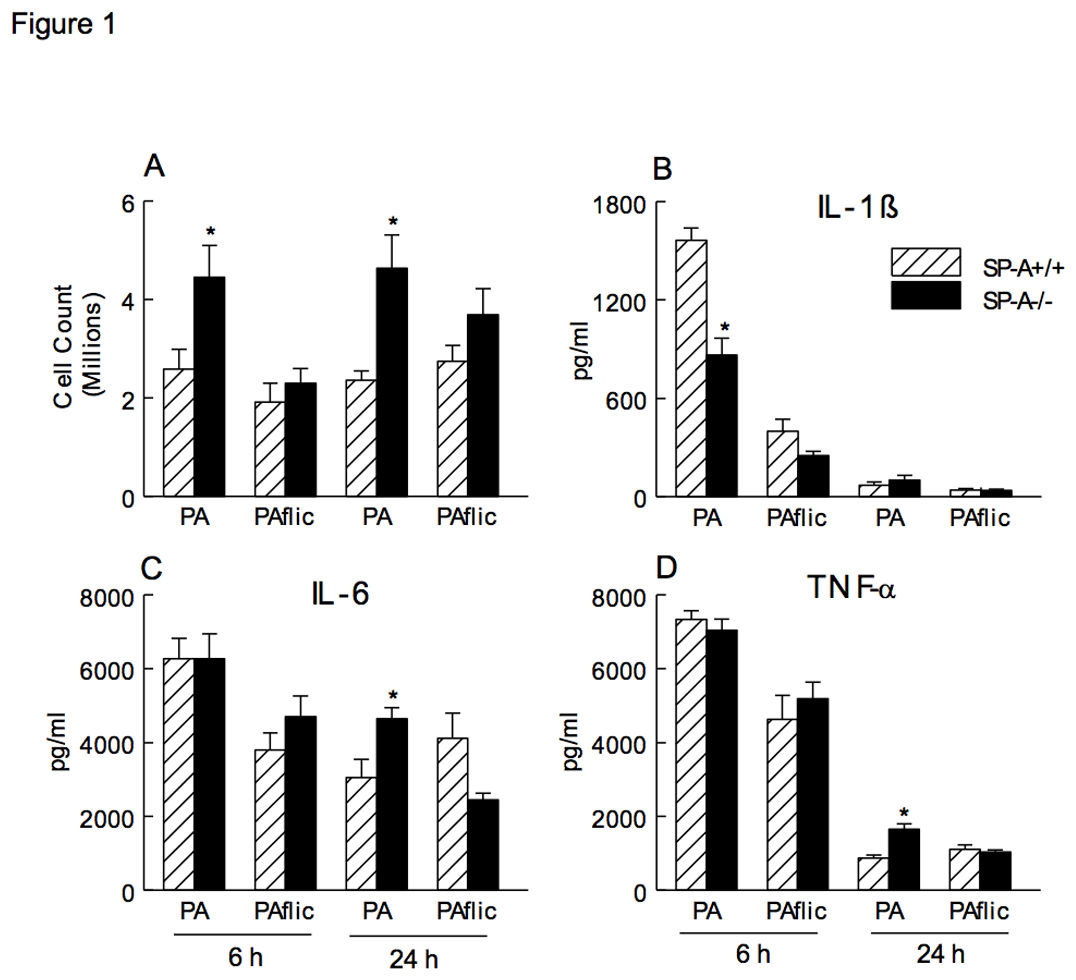
Increased total cell counts and pro-inflammatory cytokines in BAL fluid from SP-A -/- mice. Lung cells were recovered by BAL, stained with trypan blue and counted under light microscopy. Total cell counts were increased in the lung of SP-A-/- (closed bar) compared to SP-A+/+ (hatched bar) mice 6 (left panels) and 24 hours (right panels) following intra-tracheal infection with PAK. In contrast, total cell counts in the lung were similar between SP-A-/- and SP-A+/+ mice infected with *fliC* (A). IL-1β was decreased in the lung of SP-A-/- mice 6 hours following PAK infection and no difference was observed in IL-1β between SP-A-/- and SP-A+/+ mice following *fliC* infection (B). IL-6 and TNF-α were increased in the lung of SP-A-/- mice at 24 hours with PA infection, however there was no statistical difference in IL-6 or TNF-α between SP-A-/- and SP-A+/+ mice following infection with *fliC* (C, D). Data are mean ± SEM with n = 8 mice per group combined from 2 or more experiments, *p<0.05 compared to SP-A+/+ mice.

To identify SP-A specific regulation of flagella-provoked inflammation, the pulmonary inflammatory response was assessed following intra-tracheal instillation of PAK and *fliC* bacteria into SP-A+/+ and SP-A-/- mice. The intra-tracheal dose of PAK and *fliC* mutant for this study (10^8^ cfu) was determined based on previous studies [4]. Intra-tracheal administration of bacteria was well-tolerated and all animals survived the 24 hour study period. In SP-A-/- mice, increased number of cells were observed in bronchoalveolar lavage (BAL) fluid 6 and 24 hours after PAK infection. Prior studies of similar design have shown that > 95% of BAL cells are macrophages [15]. In contrast, the cell numbers were similar between SP-A+/+ and SP-A-/- mice infected with *fliC* (Figure 1A). Infection with PAK significantly increased the pro-inflammatory cytokines TNF-α and IL-6 in BAL fluid from SP-A-/- compared to SP-+/+ mice 24 hours after infection. In contrast, no significant difference was observed for TNF-α or IL-6 in the lungs following *fliC* infection (Figure 1C, D). IL-1β was decreased in the lung of SP-A-/- compared to SP-A+/+ mice 6 hours following PA infection and no difference was observed at 24 hours (Figure 1B). Similar to TNF-α and IL-6, IL-1β was not different when the mice were infected with *fliC*. 

### SP-A Binds and Enhances Phagocytosis of Flagellin

**Figure 2:**
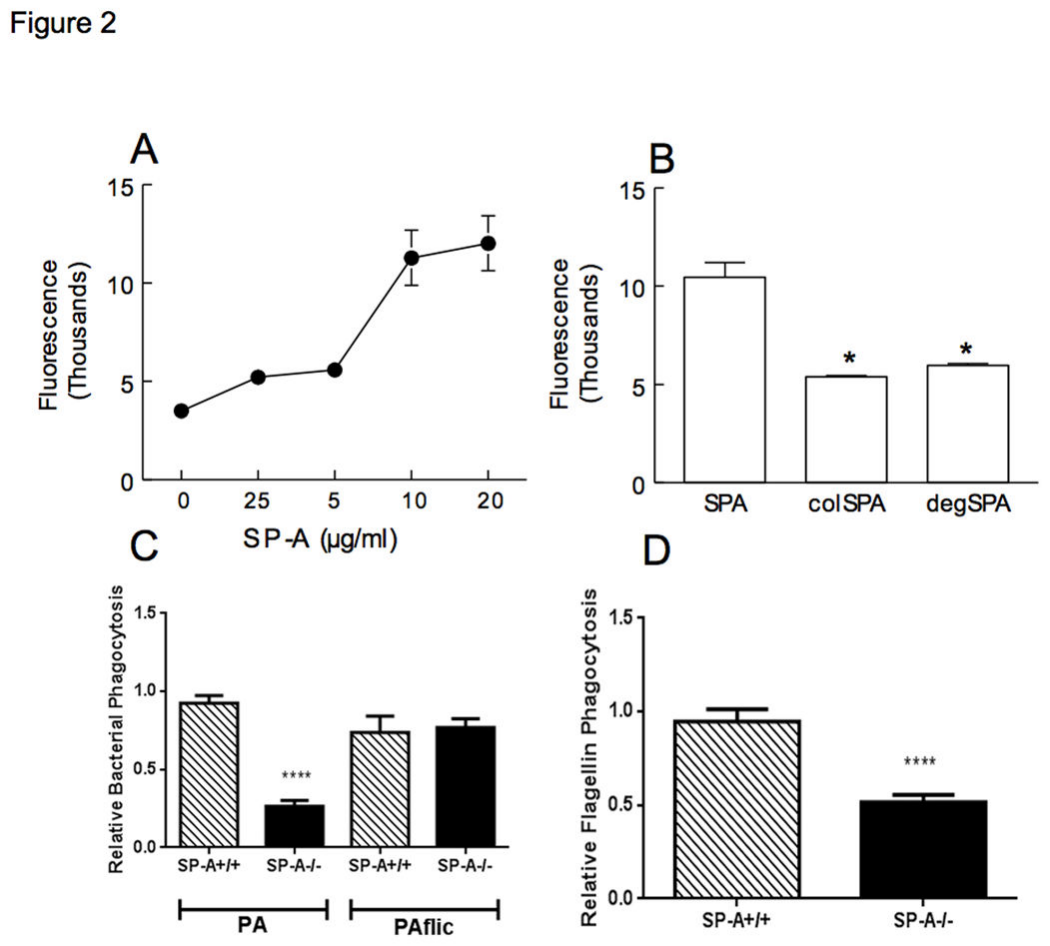
SP-A Binds Flagellin and Enhances Macrophage Phagocytosis. Fluorescently labeled SP-A bound to flagellin in a dose dependent manner (A). Binding of SP-A to flagellin was reduced in the absence of the collagen-like domain (colSPA) or the asparagine-linked carbohydrate moieties of SP-A (degSPA) (B); p<0.05 when compared to undigested SP-A. Phagocytosis of wild type PA was reduced, however phagocytosis of the *fliC* strain was similar between SP-A+/+ and SP-A-/- macrophages (C). Phagocytosis of flagellin by SP-A-/- alveolar macrophages was decreased compared to SP-A+/+ mice (D). Values for panel C were obtained from n=8 mice in each case combined from 2 experiments. Values in panel D were obtained from 4 mice combined from 2 experiments. One value for the SP-A+/+ mice in each experiment was normalized to 1, and other values are expressed relative to this normalization; p<0.0001 relative to the SP-A+/+ sample in each comparison by student’s t-test.

To determine whether SP-A interacts with flagellin a direct binding assay was performed. Flagellin (20µg/ml) was bound to a plate and FITC-labeled SP-A was added at increasing concentrations. SP-A bound to flagellin in a dose dependent manner (Figure 2A). The primary structure of SP-A is characterized by four sequential domains including a short N-terminal segment, a collagen-like region, a hydrophobic neck domain and a globular C-terminal carbohydrate recognition domain. Removal of the asparagine-linked carbohydrate moieties or the collagen-like region from SP-A both diminished binding to flagellin (Figure 2B). We conclude that SP-A binding to flagellin is dependent on both the collagen-like and N-linked sugar domains of SP-A.

The number of PAK ingested by alveolar macrophages, as assessed by flow cytometry, were decreased in SP-A-/- compared to SP-A+/+ mice, however no difference in phagocytosis was observed for *fliC* (Figure 2C). Phagocytosis of flagellin alone was assessed by flow cytometry to determine if the difference observed in the uptake of PAK and *fliC* was due to the presence of flagella. Phagocytosis of flagellin was reduced by 50% when incubated with alveolar macrophages isolated from SP-A-/- mice *ex vivo* (Figure 2D). This data indicates that SP-A binds and enhances phagocytosis of flagellin.

### SP-A Binds Scavenger Receptors to Enhance Uptake of Opsonized Particles

**Figure 3:**
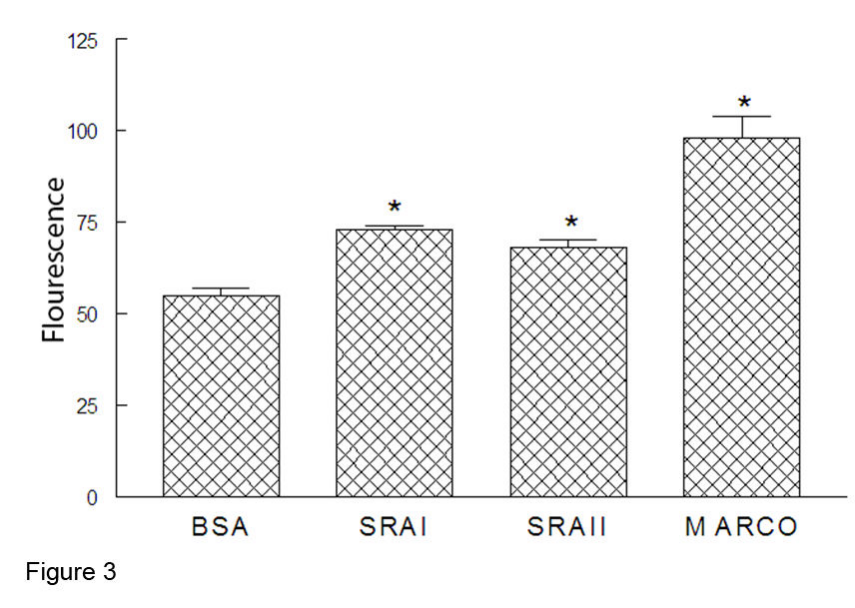
SP-A binds Scavenger Receptors. FITC-labeled SP-A was incubated with immobilized bovine serum albumin (BSA) or purified SRA-I, SRA-II, or MARCO. SP-A was able to bind to all three scavenger receptors suggesting a means to enhance phagocytosis of flagellated bacteria; n=3, *p<0.05 when compared to BSA control.

These data suggested that SP-A enhanced uptake of bacteria via flagellin binding, but the mechanism of how this happens was not clear. We speculated that SP-A could bind to scavenger receptors to facilitate uptake. Figure 3 demonstrates that purified SP-A binds to both macrophage scavenger receptor A I and II (SRA I and II) as well as to the macrophage receptor with collagenous structure (MARCO). Thus, these data suggest that SP-A, via its ability to bind to both flagellin and scavenger receptors facilitates bacterial phagocytosis.

### SP-A Increased IL-1β from Macrophages Stimulated with Flagellin

**Figure 4:**
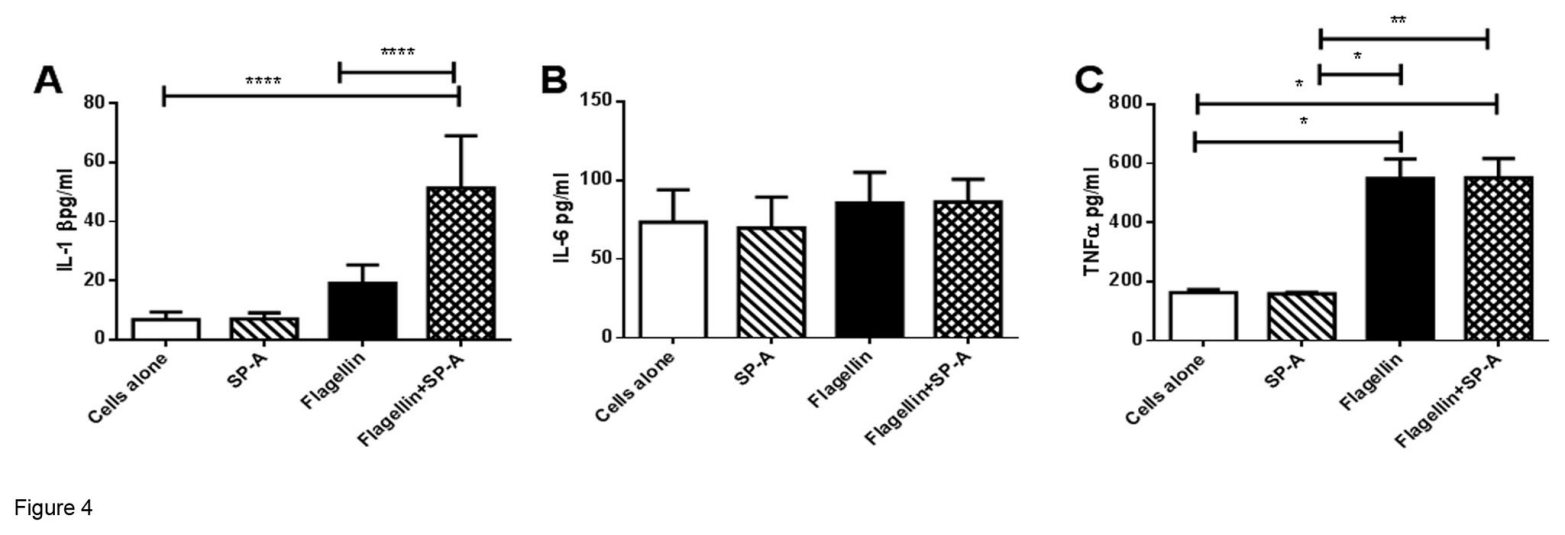
SP-A Enhanced Flagellin Stimulated IL-1β Production. Concentrations of IL-1β, IL-6 and TNF-α, were assessed in the media of MH-s cells following stimulation with SP-A alone, flagellin or flagellin with SP-A. IL-1β, but not IL-6 or TNF-α, production was enhanced by flagella with SP-A after the 60min stimulation. Data is expressed as pg/ml and represent mean ±SEM, with n=6-10 separate samples/group. *p<0.05, *p<0.01, ****p<0.0001 when analyzed by Kuskal-Walis ANOVA followed by Dunn’s multiple comparison test.

Since IL-1β was reduced and TNF-α and IL-6 were increased *in vivo* with intra-tracheal infection of PAK and *fliC* in SP-A-/- mice, studies were performed on stimulated murine macrophage (MH-s) cells to determine the specific effects of SP-A on the macrophage response to flagellin. MH-s cells were stimulated with SP-A alone, flagellin alone or flagellin plus SP-A for 1 h and pro-inflammatory cytokines were determined in the media. SP-A did not alter the production of TNF-α or IL-6 by MH-s cells stimulated with flagellin during this shorter exposure (Figure 4B, C). In contrast, SP-A augmented the production of IL-1β by flagellin stimulated MH-s cells (Figure 4A). These findings are consistent with the PA infected mouse model revealing decreased IL-β in the lung in the absence of SP-A.

### SP-A Does Not Enhance TLR-5 Signaling in MH-s Cells Stimulated with Flagellin

**Figure 5:**
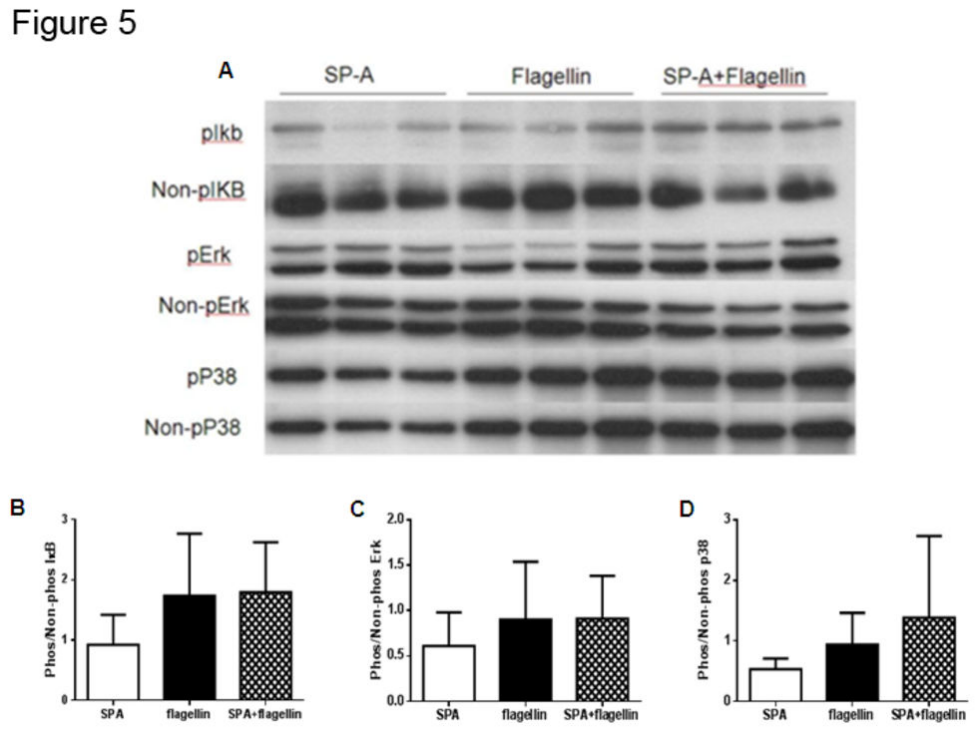
TLR5 Signaling is not Enhanced by SP-A Interactions with Flagellin. Immunoblot was used to compare the phosphorylated and non-phosphorylated TLR-5 signaling pathway including IκBα, ERK and p38 in cell lysates from MH-s cells following stimulation with SP-A, flagellin or flagellin with SP-A. Blot in panel A is a representative experiment. Densitometry was performed on three experiments with multiple replicates and expressed as the phosphorylated divided by the non-phosphorylated form (B, C, D). There were no significant differences between any of the stimulations. Data shown are from 20 minutes of stimulation, but there were also no differences between groups noted following 10, or 60 minutes of stimulation as well (data not shown).

To determine the specific role of SP-A in flagellin signaling responses in macrophages, MH-s cells were stimulated with SP-A, flagellin or flagellin with SP-A for 20 minutes and Western blot analysis was performed for active phosphorylated and inactive non-phosphorylated kinases p38, ERK, and IκBα (Figure 5A). Densitometry was performed on the Western blots and the results were reported as the phosphorylated protein divided by the non-phosphorylated proteins (Figure 5B, C, D). MH-s cells stimulated with flagellin expressed similar p38, ERK, and IκBα as cells stimulated with flagellin with SP-A. These findings suggest that the flagellin of PA does not contribute to the overproduction of TNF-α and IL-6 in the lungs of SP-A-/- mice 24 h following infection with PA.

### SP-A Enhanced Flagellin Stimulated Production of IL-1β Is Caspase-1 Dependent

**Figure 6:**
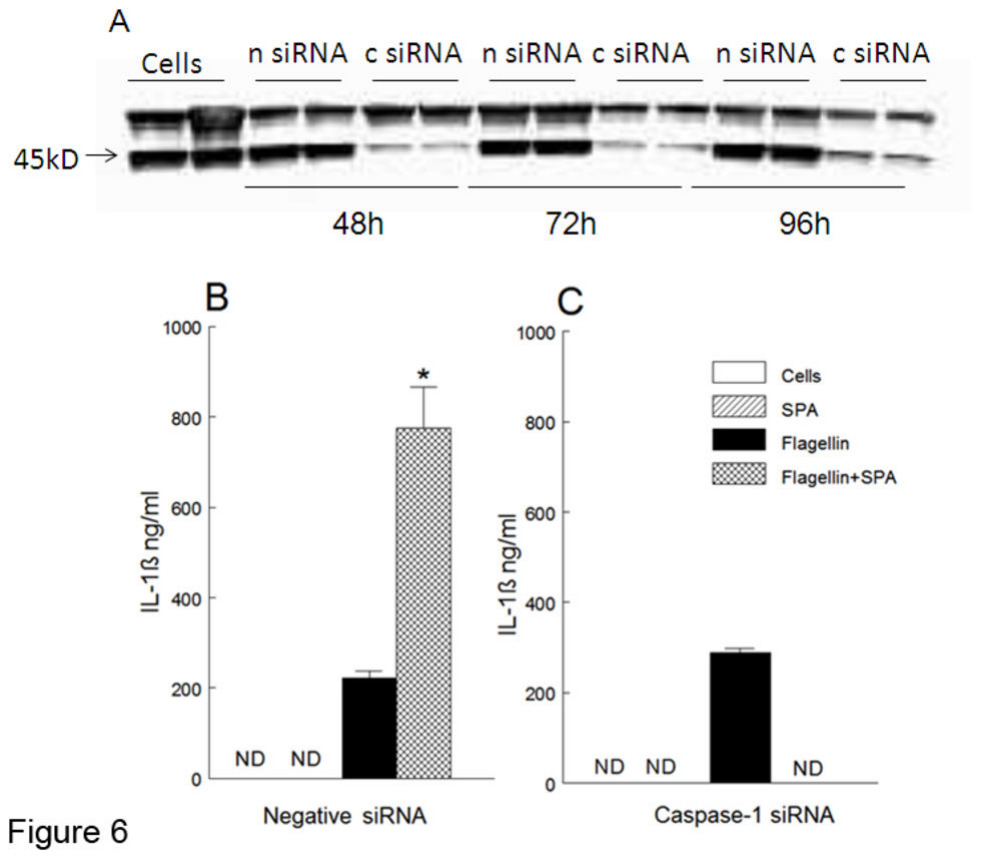
Silencing of Caspase-1 Reduced the SP-A Enhanced IL-1β Production Following Flagellin Stimulation. Caspase-1 was silenced in MH-s cells using siRNA (c siRNA) and negative siRNA (n siRNA). Reduced caspase-1 (45kD band) was observed by immunoblot 48, 72 and 96 following cell transfection of caspase-1 siRNA (A). SP-A enhanced IL-1β production following flagellin stimulation which was decreased when caspase-1 activity was reduced (B, C).

 Since SP-A does not appear to enhance TLR-5 signaling it was hypothesized that SP-A enhances flagellin-stimulated IL-1β production through the caspase-1 activating inflammasome pathway. MH-s cells were transiently transfected with siRNA for caspase-1 (c si RNA) or a negative control siRNA (n si RNA). Cell viability as determined microscopically following trypan blue staining was >80% in all transfections. Protein levels were determined by Western blot analysis revealing a decrease in the 45 kDa caspase-1 protein at 48, 72 and 96 hours in c si RNA treated cells (Figure 6A). Based on the reduced protein levels observed, at 48 hours the transfected MH-s cells were stimulated with SP-A, flagellin or flagellin with SP-A. Similar to the previous results without transfection, SP-A enhanced flagellin stimulated IL-1β production by MH-s cells transfected with n siRNA (Figure 6B). However, when caspase-1 was silenced, there was complete reversal of the SP-A enhancement of flagellin stimulated IL-1β production (Figure 6C). 

## Discussion

Surfactant protein A (SP-A) enhances pulmonary clearance of *Pseudomonas aeruginosa* (PA) [4]. Consistent with current evidence, intra-tracheal infection of wild type flagellated PA resulted in increased inflammatory cell recruitment and pro-inflammatory cytokines IL-6 and TNF-α in the lungs of SP-A-/- mice which was not observed with mutant PA lacking flagella. In contrast, and not demonstrated in prior studies, there was decreased expression of IL-1β in the lungs of SP-A-/- mice intra-tracheally infected with wild type PA and an increase of IL-1β when macrophages were stimulated *in vitro* with flagellin in the presence of SP-A. This uniquely suggests that SP-A enhances macrophage IL-1β production with flagella stimulation. SP-A binds flagellin directly and enhances its phagocytosis, likely via enhancing the interactions with SRA and MARCO. Silencing of caspase-1 in macrophages *in vitro* reduced the SP-A enhanced IL-1β production following flagellin stimulation supporting a new role for the inflammasome in SP-A regulation of inflammation. 

Following intra-tracheal installation of flagellated wild type PA, markers of inflammation including macrophage-lineage inflammatory cells, IL-6 and TNF-α were increased in the lungs of SP-A-/- mice after 24 hours of exposure (Figure 1). No statistically significant differences in cytokine production were observed with infection from the *fliC* mutant. The elevated IL-6 and TNF-α observed *in vivo* (Figure 1) was not observed in the *in vitro* studies (Figure 4) likely due to a difference in incubation time (1 h versus 24 h). Previous studies revealed a pro-inflammatory response in the lungs of SP-A-/- mice following infection with bacteria including gram negative *Haemophilus influenzae* and gram positive group B streptococcus [2]. Pulmonary clearance of mucoid PA is impaired in the lungs of SP-A-/- mice and is also associated with increased pro-inflammatory cytokines, including TNF-α and IL-6, in the lung compared to wild type mice [4]. Honko et al. observed elevated levels of TNF-α and IL-6 in the murine lung after intra-tracheal flagellin administration [16]. The expression of TNF-α and IL-6 is mediated through the NF-κB pathway and SP-A confers anti-inflammatory immune regulation by increasing the accumulation of IκB-α, the predominant inhibitor of NF-κB [17,18]. In AMs, SP-A pretreatment caused a marked inhibition of lipopolysaccharide (LPS)-induced NF-κB activation [18]. SP-A has been reported to decrease TNF-α expression from rat alveolar macrophages stimulated with LPS [19,20] and inhibits the production of several pro-inflammatory cytokines, including TNF-α and IL-1β from human alveolar macrophages stimulated with *Candida albicans* [21]. The current, as well as previous, studies support an anti-inflammatory role of SP-A in the lung following microbial infection.

SP-A bound flagellin in the presence of calcium in a concentration dependent manner. SP-A is a calcium dependent lectin and binding to macrophages, LPS, Toll-like receptor 4 and complement receptor 3 have also been shown to be dependent on calcium [22–24]. Binding of SP-A to CD-14 is independent of calcium and has been shown to occur through the neck region of SP-A [25]. In the current study, SP-A binding to flagellin required calcium suggesting the lectin domain in this interaction [26]. To further clarify the interaction of SP-A and flagellin, the asparagine-linked carbohydrate moieties or the collagen-like region from SP-A were removed using N-glycosidase or collagenase, which diminished binding to flagellin. This suggests binding to be occurring at the collagenase resistant fragment, which consists of the CRD and neck region of SP-A, and is consistent with previous findings of interactions with SP-A to conserved pathogen-associated molecular patterns (PAMPs), such as rough LPS and lipid A [19]. We conclude that SP-A binding to flagellin is dependent on both the collagen like and N-linked sugar domains of SP-A.

SP-A increased phagocytosis of wild type PA but not mutant PA lacking flagella *in vivo* and also enhanced phagocytosis of flagellin *in vitro*. SP-A increases phagocytosis of serum-opsonized *Staphylococcus aureus* by alveolar macrophages [27] and increases serum independent phagocytosis of *Escherichia coli*, *Pseudomonas aeruginosa* and *Staphylococcus aureus* [28]. Ingestion of bacteria by alveolar macrophages is important in the early elimination of some bacterial species from the lung and SP-A can enhance phagocytosis by macrophages [28,29]. Our current studies confirm that SP-A can mediate binding to the scavenger receptors SRAI, SRAII and MARCO (Figure 3). Additionally, SP-A stimulates directional actin polymerization specifically in alveolar macrophages [30] and SP-A induction of phagocytosis utilizes pathways similar to those used by IgG, dependent on tyrosine kinases, protein kinase C and actin polymerization but not on microtubule activity [31]. Delayed ingestion of PA by alveolar macrophages may be one of the factors that allow PA infection to be established in the lung. Intra-tracheal administration of SP-A increased phagocytosis of group B streptococci (GBS) in SP-A-/- mice suggesting that SP-A plays an immediate and direct role in the clearance of GBS by directly binding to these organisms and acting as an opsonin [32]. Previous studies report that flagellin is important for non-opsonic phagocytosis of PA [8]. Similar to the current results, Zhang et al. determined that SP-A increased macrophage phagocytosis of a wild type flagellated PA strain but not the mutant *flgE* PA lacking flagella function [7]. In the absence of SP-A, macrophage phagocytosis of wild type PA is decreased and the flagella of PA are important for SP-A enhanced phagocytosis.

Flagellin signals through the TLR-5 pathway in macrophages but no further increase in the TLR-5 signaling pathway was observed in the presence of SP-A. Flagellin interacts extracellularly with TLR-5 [9] and intracellularly with the inflammasome [11] to produce IL-1β. Inhibitor of kappa B (IκB), extracellular regulated kinase (ERK) and p38 MAP kinase are all intermediate signaling proteins phosphorylated downstream when flagellin binds to TLR-5. Descamps et al. suggested that TLR-5 signaling is critical for flagella-induced IL-1β production and ultimate killing of PA by alveolar macrophages [33]. However, following infection of alveolar macrophages from TLR-5-/- mice with flagellated salmonella and flagellated PA, IL-1β production was still detectable [10,12]. This suggests the activation of an alternative pathway for IL-1β production, such as the inflammasome. SP-A inhibits macrophage production of TNF-α following stimulation with LPS independent of TLR-4 (the LPS binding receptor) since TNF-α production was also decreased by SP-A in TLR4-deficient mutant macrophages [34], suggesting other pathways in which SP-A regulates inflammation. Since IL-1β levels increased after flagellin stimulation, independent of the TLR5 signal transduction pathway, SP-A may be enhancing IL-1β expression through the inflammasome pathway; a conclusion supported by our current studies.

SP-A augmented the flagellin stimulated production of IL-1β by macrophages. This result was consistent with the *in vivo* observation of decreased IL-1β expression in the lungs of SP-A-/- mice exposed to flagella. Although TNF-α, IL-6 and IL-1β are all pro-inflammatory cytokines they appear to be differentially regulated by SP-A in the lung following flagella stimulation. SP-A did not enhance flagellin stimulated IL-1β expression when caspase-1 was silenced in macrophages supporting a role for SP-A through the inflammasome pathway. It is possible that SP-A serves as an opsonin enhancing intracellular flagellin through phagocytosis independent of the TLR-5 receptor rendering the flagellin available to intracellularly interact with the inflammasome to generate IL-1β. SP-A binds several phagocytic receptors including CD14, complement receptor 3 and scavenger receptor [22,25,35] that may provide an entry point for SP-A opsonized flagella into the phagosome. We provided evidence in the current study that SP-A directly binds flagellin as well as the two most common scavenger receptors present on alveolar macrophages [36]. This likely facilitates bacterial phagocytosis. How the bacteria or flagellin escape the phagosome to enter the cytoplasm is still unclear. The cytosolic nucleotide-binding and oligomerization domain (NOD) like receptors (NLRs) would then be able to recognize the flagellin in the cytosol [37]. Thus, a conceivable model to explain these results is that SP-A binds and enhances phagocytosis of flagella stimulating IL-1β production through the inflammasome pathway.

SP-A enhanced flagellin stimulated IL-1β production may be important for the clearance of PA. Pulmonary clearance of intra-tracheally administered PA is reduced in SP-A-/- mice associated with increased lung inflammation [4]. Though IL-1β may be destructive to the lung, it also participates in the killing of bacteria by contributing to the maturation and acidification of the phagosome [33,38]. In addition, mice lacking the IL-1 receptor (IL-1R1-/-) were unable to kill PA, providing additional support for a role for IL-1β in killing PA [33]. Also, IL-1β stimulation results in the generation of inducible nitric oxide synthetase (iNOS) from cytokine activated macrophages [39] resulting in the production of nitric oxide (NO). SP-A has directly been implicated in the production of NO [13] and NO has significant bactericidal effects against PA [14]. Therefore, SP-A may enhance clearance of PA, in part, by enhanced flagellin mediated bacterial phagocytosis and increased killing of the bacteria through IL-1β. Additionally, by increasing flagella substrate intracellularly, SP-A may indirectly be driving NLRC4 inflammasome function, resulting in increased activation of caspase-1. Byrne et al. demonstrated increased macrophage self-degradation, known as autophagy, occurring with intracellular flagellated *Legionella pneumophila* activating the NLRC4 inflammasome, increasing clearance of the bacteria in addition to raising the occurrence threshold of proinflammatory cell death known as pyroptosis [40]. Supporting NLRC4 influenced bacterial clearance, *Salmonella enterica* promotes its own survival and prevents the clearance of infection by down regulating NLRC4 in B-cells [41]. Therefore, SP-A may be promoting the clearance of PA infection by both the activation of the inflammasome and the production of IL-1β.

In summary, the present study supports a new role for SP-A in binding and enhancing the clearance of flagellin, mediated in part by enhanced IL-1β production likely through the inflammasome pathway. We speculate that reduction of SP-A in the lungs of patients with inflammatory lung diseases such as bronchopulmonary dypslasia (BPD), cystic fibrosis and ventilator associate pneumonia increases the susceptibility to pneumonia and sepsis.

## References

[1] Wu H, Kuzmenko A, Wan S, Schaffer L, Weiss A et al. (2003) Surfactant proteins A and D inhibit the growth of Gram-negative bacteria by increasing membrane permeability. J Clin Invest 111: 1589-1602. doi:10.1172/JCI16889. PubMed: 12750409. 12750409 PMC155045

[2] LeVine AM, Whitsett JA, Gwozdz JA, Richardson TR, Fisher JH et al. (2000) Distinct effects of surfactant protein A or D deficiency during bacterial infection on the lung. J Immunol 165: 3934-3940. PubMed: 11034401. 11034401 10.4049/jimmunol.165.7.3934

[3] Mariencheck WI, Savov J, Dong Q, Tino MJ, Wright JR (1999) Surfactant protein A enhances alveolar macrophage phagocytosis of a live, mucoid strain of P. aeruginosa. Am J Physiol 277: L777-L786. PubMed: 10516219. 10516219 10.1152/ajplung.1999.277.4.L777

[4] LeVine AM, Kurak KE, Bruno MD, Stark JM, Whitsett JA et al. (1998) Surfactant protein-A-deficient mice are susceptible to Pseudomonas aeruginosa infection. Am J Respir Cell Mol Biol 19: 700-708. doi:10.1165/ajrcmb.19.4.3254. PubMed: 9761768. 9761768

[5] Feldman M, Bryan R, Rajan S, Scheffler L, Brunnert S et al. (1998) Role of flagella in pathogenesis of Pseudomonas aeruginosa pulmonary infection. Infect Immun 66: 43-51. PubMed: 9423837. 9423837 10.1128/iai.66.1.43-51.1998 PMC107856

[6] Ramphal R, Balloy V, Jyot J, Verma A, Si-Tahar M et al. (2008) Control of Pseudomonas aeruginosa in the lung requires the recognition of either lipopolysaccharide or flagellin. J Immunol 181: 586-592. PubMed: 18566425. 18566425 10.4049/jimmunol.181.1.586 PMC2504754

[7] Zhang S, McCormack FX, Levesque RC, O'Toole GA, Lau GW (2007) The flagellum of Pseudomonas aeruginosa is required for resistance to clearance by surfactant protein A. PLOS ONE 2: e564. doi:10.1371/journal.pone.0000564. PubMed: 17593964. 17593964 PMC1891440

[8] Mahenthiralingam E, Speert DP (1995) Nonopsonic phagocytosis of Pseudomonas aeruginosa by macrophages and polymorphonuclear leukocytes requires the presence of the bacterial flagellum. Infect Immun 63: 4519-4523. PubMed: 7591095. 7591095 10.1128/iai.63.11.4519-4523.1995 PMC173644

[9] Raoust E, Balloy V, Garcia-Verdugo I, Touqui L, Ramphal R et al. (2009) Pseudomonas aeruginosa LPS or flagellin are sufficient to activate TLR-dependent signaling in murine alveolar macrophages and airway epithelial cells. PLOS ONE 4: e7259. doi:10.1371/journal.pone.0007259. PubMed: 19806220. 19806220 PMC2752798

[10] Franchi L, Amer A, Body-Malapel M, Kanneganti TD, Ozören N et al. (2006) Cytosolic flagellin requires Ipaf for activation of caspase-1 and interleukin 1beta in salmonella-infected macrophages. Nat Immunol 7: 576-582. doi:10.1038/ni1346. PubMed: 16648852. 16648852

[11] Miao EA, Alpuche-Aranda CM, Dors M, Clark AE, Bader MW et al. (2006) Cytoplasmic flagellin activates caspase-1 and secretion of interleukin 1beta via Ipaf. Nat Immunol 7: 569-575. doi:10.1038/ni1344. PubMed: 16648853. 16648853

[12] Franchi L, Stoolman J, Kanneganti TD, Verma A, Ramphal R et al. (2007) Critical role for Ipaf in Pseudomonas aeruginosa-induced caspase-1 activation. Eur J Immunol 37: 3030-3039. doi:10.1002/eji.200737532. PubMed: 17935074. 17935074

[13] Korfhagen TR, Bruno MD, Ross GF, Huelsman KM, Ikegami M et al. (1996) Altered surfactant function and structure in SP-A gene targeted mice. Proc Natl Acad Sci U S A 93: 9594-9599. doi:10.1073/pnas.93.18.9594. PubMed: 8790375. 8790375 PMC38473

[14] Haagsman HP, Hawgood S, Sargeant T, Buckley D, White RT et al. (1987) The major lung surfactant protein, SP 28-36, is a calcium-dependent, carbohydrate-binding protein. J Biol Chem 262: 13877-13880. PubMed: 2820982. 2820982

[15] LeVine AM, Bruno MD, Huelsman KM, Ross GF, Whitsett JA et al. (1997) Surfactant protein A-deficient mice are susceptible to group B streptococcal infection. J Immunol 158: 4336-4340. PubMed: 9126996. 9126996

[16] Honko AN, Mizel SB (2004) Mucosal administration of flagellin induces innate immunity in the mouse lung. Infect Immun 72: 6676-6679. doi:10.1128/IAI.72.11.6676-6679.2004. PubMed: 15501801. 15501801 PMC523048

[17] Moulakakis C, Adam S, Seitzer U, Schromm AB, Leitges M et al. (2007) Surfactant protein A activation of atypical protein kinase C zeta in IkappaB-alpha-dependent anti-inflammatory immune regulation. J Immunol 179: 4480-4491. PubMed: 17878344. 17878344 10.4049/jimmunol.179.7.4480

[18] Wu Y, Adam S, Hamann L, Heine H, Ulmer AJ et al. (2004) Accumulation of inhibitory kappaB-alpha as a mechanism contributing to the anti-inflammatory effects of surfactant protein-A. Am J Respir Cell Mol Biol 31: 587-594. doi:10.1165/rcmb.2004-0003OC. PubMed: 15308505. 15308505

[19] Sano H, Sohma H, Muta T, Nomura S, Voelker DR et al. (1999) Pulmonary surfactant protein A modulates the cellular response to smooth and rough lipopolysaccharides by interaction with CD14. J Immunol 163: 387-395. PubMed: 10384140. 10384140

[20] McIntosh JC, Mervin-Blake S, Conner E, Wright JR (1996) Surfactant protein A protects growing cells and reduces TNF-alpha activity from LPS-stimulated macrophages. Am J Physiol 271: L310-L319. PubMed: 8770070. 8770070 10.1152/ajplung.1996.271.2.L310

[21] Rosseau S, Hammerl P, Maus U, Günther A, Seeger W et al. (1999) Surfactant protein A down-regulates proinflammatory cytokine production evoked by Candida albicans in human alveolar macrophages and monocytes. J Immunol 163: 4495-4502. PubMed: 10510392. 10510392

[22] Gil M, McCormack FX, Levine AM (2009) Surfactant protein A modulates cell surface expression of CR3 on alveolar macrophages and enhances CR3-mediated phagocytosis. J Biol Chem 284: 7495-7504. doi:10.1074/jbc.M808643200. PubMed: 19155216. 19155216 PMC2658045

[23] Guillot L, Balloy V, McCormack FX, Golenbock DT, Chignard M et al. (2002) Cutting edge: the immunostimulatory activity of the lung surfactant protein-A involves Toll-like receptor 4. J Immunol 168: 5989-5992. PubMed: 12055204. 12055204 10.4049/jimmunol.168.12.5989

[24] Pikaar JC, Voorhout WF, van Golde LM, Verhoef J, Van Strijp JA et al. (1995) Opsonic activities of surfactant proteins A and D in phagocytosis of gram-negative bacteria by alveolar macrophages. J Infect Dis 172: 481-489. doi:10.1093/infdis/172.2.481. PubMed: 7622892. 7622892

[25] Sano H, Chiba H, Iwaki D, Sohma H, Voelker DR et al. (2000) Surfactant proteins A and D bind CD14 by different mechanisms. J Biol Chem 275: 22442-22451. doi:10.1074/jbc.M001107200. PubMed: 10801802. 10801802

[26] Shang F, Rynkiewicz MJ, McCormack FX, Wu H, Cafarella TM et al. (2011) Crystallographic complexes of surfactant protein A and carbohydrates reveal ligand-induced conformational change. J Biol Chem 286: 757-765. doi:10.1074/jbc.M110.175265. PubMed: 21047777. 21047777 PMC3013034

[27] van Iwaarden F, Welmers B, Verhoef J, Haagsman HP, van Golde LM (1990) Pulmonary surfactant protein A enhances the host-defense mechanism of rat alveolar macrophages. Am J Respir Cell Mol Biol 2: 91-98. doi:10.1165/ajrcmb/2.1.91. PubMed: 2306370. 2306370

[28] Manz-Keinke H, Plattner H, Schlepper-Schäfer J (1992) Lung surfactant protein A (SP-A) enhances serum-independent phagocytosis of bacteria by alveolar macrophages. Eur J Cell Biol 57: 95-100. PubMed: 1639094. 1639094

[29] Kabha K, Schmegner J, Keisari Y, Parolis H, Schlepper-Schaeffer J et al. (1997) SP-A enhances phagocytosis of Klebsiella by interaction with capsular polysaccharides and alveolar macrophages. Am J Physiol 272: L344-L352. PubMed: 9124386. 9124386 10.1152/ajplung.1997.272.2.L344

[30] Tino MJ, Wright JR (1999) Surfactant proteins A and D specifically stimulate directed actin-based responses in alveolar macrophages. Am J Physiol 276: L164-L174. PubMed: 9887069. 9887069 10.1152/ajplung.1999.276.1.L164

[31] Schagat TL, Tino MJ, Wright JR (1999) Regulation of protein phosphorylation and pathogen phagocytosis by surfactant protein A. Infect Immun 67: 4693-4699. PubMed: 10456918. 10456918 10.1128/iai.67.9.4693-4699.1999 PMC96796

[32] LeVine AM, Kurak KE, Wright JR, Watford WT, Bruno MD et al. (1999) Surfactant protein-A binds group B streptococcus enhancing phagocytosis and clearance from lungs of surfactant protein-A-deficient mice. Am J Respir Cell Mol Biol 20: 279-286. doi:10.1165/ajrcmb.20.2.3303. PubMed: 9922219. 9922219

[33] Descamps D, Le Gars M, Balloy V, Barbier D, Maschalidi S, et al. (2012) Toll-like receptor 5 (TLR5), IL-1beta secretion, and asparagine endopeptidase are critical factors for alveolar macrophage phagocytosis and bacterial killing. Proc Natl Acad Sci U S A 109: 1619-1624 10.1073/pnas.1108464109 PMC3277124 22307620

[34] Alcorn JF, Wright JR (2004) Surfactant protein A inhibits alveolar macrophage cytokine production by CD14-independent pathway. Am J Physiol Lung Cell Mol Physiol 286: L129-L136. PubMed: 12959932. 12959932 10.1152/ajplung.00427.2002

[35] Sever-Chroneos Z, Krupa A, Davis J, Hasan M, Yang CH et al. (2011) Surfactant protein A (SP-A)-mediated clearance of Staphylococcus aureus involves binding of SP-A to the staphylococcal adhesin eap and the macrophage receptors SP-A receptor 210 and scavenger receptor class A. J Biol Chem 286: 4854-4870. doi:10.1074/jbc.M110.125567. PubMed: 21123169. 21123169 PMC3039347

[36] Domingo-Gonzalez R, Katz S, Serezani CH, Moore TA, Levine AM et al. (2013) Prostaglandin E2-induced changes in alveolar macrophage scavenger receptor profiles differentially alter phagocytosis of Pseudomonas aeruginosa and Staphylococcus aureus post-bone marrow transplant. J Immunol 190: 5809-5817. doi:10.4049/jimmunol.1203274. PubMed: 23630358. 23630358 PMC3660503

[37] Halff EF, Diebolder CA, Versteeg M, Schouten A, Brondijk TH et al. (2012) Formation and structure of a NAIP5-NLRC4 inflammasome induced by direct interactions with conserved N- and C-terminal regions of flagellin. J Biol Chem 287: 38460-38472. doi:10.1074/jbc.M112.393512. PubMed: 23012363. 23012363 PMC3493891

[38] Master SS, Rampini SK, Davis AS, Keller C, Ehlers S et al. (2008) Mycobacterium tuberculosis prevents inflammasome activation. Cell Host Microbe 3: 224-232. doi:10.1016/j.chom.2008.03.003. PubMed: 18407066. 18407066 PMC3657562

[39] Kremlev SG, Umstead TM, Phelps DS (1997) Surfactant protein A regulates cytokine production in the monocytic cell line THP-1. Am J Physiol 272: L996-1004. PubMed: 9176266. 9176266 10.1152/ajplung.1997.272.5.L996

[40] Byrne BG, Dubuisson JF, Joshi AD, Persson JJ, Swanson MS (2013) Inflammasome components coordinate autophagy and pyroptosis as macrophage responses to infection. MBio 4: e00620–12 PubMed: 23404401. 23404401 10.1128/mBio.00620-12 PMC3573666

[41] Perez-Lopez A, Rosales-Reyes R, Alpuche-Aranda CM, Ortiz-Navarrete V (2013) Salmonella downregulates nod-like receptor family CARD domain containing protein 4 expression to promote its survival in B cells by preventing inflammasome activation and cell death. J Immunol 190: 1201-1209. doi:10.4049/jimmunol.1200415. PubMed: 23284055. 23284055

